# ASO Author Reflections on “Variation in Surgical Management of Stage IV Breast Cancer: Local/Institutional and Regional Geographic Considerations.”

**DOI:** 10.1245/s10434-026-20273-4

**Published:** 2026-07-19

**Authors:** Stephanie Serres, Shruti Zaveri

**Affiliations:** Department of Surgery, Division of Surgical Oncology, The University of Texas Southwestern Medical Center, Dallas, TX USA

## The Past

Indications for the surgical management of de novo stage IV breast cancer have varied over time. The historical paradigm for management of stage IV breast cancer was to focus on palliation, and locoregional treatment was reserved for patients needing symptom control in cases of bleeding, pain, and imminent skin ulceration.^1^ Starting in the early 2000s, several retrospective studies evaluated surgery with the intended goal of survival advantage in stage IV breast cancer. ^1,2^ These studies demonstrated mixed results; however, the studies showing a survival advantage were often plagued by significant biases, such as immortal time, stage migration, and selection biases. ^1,3^ More recent randomized controlled trials have largely demonstrated negligible survival advantage with surgical intervention.^4^

## The Present

Our study assesses variation in management of de novo stage IV breast cancer in a nationally representative study utilizing data from over 68,000 patients in the National Cancer Database.^5^ Among this cohort, 25.7% of patients underwent surgical management of a locoregional or distant metastatic site. Importantly, rates of locoregional surgical intervention decrease over time, aligning with the publication of the randomized controlled trials. However, even in the most contemporary timeframe, 15% of patients with stage IV breast cancer continued to undergo surgical intervention. Variation was also noted among geographic regions: the Northeast  showed the lowest rates of surgical intervention and the West coast showed the highest. Significant variation was also noted among facilities based on their type and patient volume: community cancer programs and facilities with the lowest volumes had the highest rates of operative interventions. Strikingly, at the individual institution level, the proportion of patients undergoing surgical intervention ranged from 0% to 100%.

## The Future

Decision-making for breast cancer is often complex and affected by both patient and institutional factors, such as referral patterns and tumor board dynamics. However, the observed institution-level variability from 0% to 100% is profound. Although the National Cancer Database data do not allow for assessment of the individual surgical decision-making, it seems unlikely that palliation was the surgical approach in all cases who pursued operative intervention at institutions where 100% of patients with stage IV disease underwent surgery. As low-volume institutions were more likely to provide operative intervention, the findings highlight the need for effective and efficient distribution of clinical trial findings to a broad range of facilities, particularly those that may not treat high volumes of breast cancer. Effective distribution of evidence is particularly important in breast cancer treatment, as systemic therapies improve and the treatment landscape is constantly changing.
